# The Role of Photodynamic Therapy in the Treatment of Vulvar Intraepithelial Neoplasia

**DOI:** 10.3390/biomedicines6010013

**Published:** 2018-02-02

**Authors:** Giulio Tosti, Anna Daniela Iacobone, Eleonora Petra Preti, Sabina Vaccari, Alessia Barisani, Elisabetta Pennacchioli, Carmen Cantisani

**Affiliations:** 1 Division of Melanoma, Soft Tissue Sarcomas and Rare Tumors, Istituto Europeo di Oncologia, Via G. Ripamonti 437, 20141 Milano, Italy; elisabetta.pennacchioli@ieo.it; 2 Preventive Gynecology Unit, Istituto Europeo di Oncologia, 20141 Milano, Italy; AnnaDaniela.Iacobone@ieo.it (A.D.I.); eleonora.preti@ieo.it (E.P.P.); 3 Unit of Dermatology, Department of Experimental, Diagnostic and Specialty Medicine, Policlinico Sant’Orsola-Malpighi, 40138 Bologna, Italy; sabina.vaccari@aosp.bo.it (S.V.); alessiabarisani@gmail.com (A.B.); 4 Department of Dermatology, Policlinico Umberto I, Sapienza University of Rome, 00185 Rome, Italy; carmencantisanister@gmail.com

**Keywords:** vulvar intraepithelial neoplasia, vulvar intraepithelial neoplasia (VIN), photodynamic therapy, PDT, vulvar intraepithelial neoplasia treatment, VIN treatment

## Abstract

Background: vulvar intraepithelial neoplasia is a non-invasive precursor lesion found in 50–70% of patients affected by vulvar squamous cell carcinoma. In the past, radical surgery was the standard treatment for vulvar intraepithelial neoplasia, however, considering the psychological and physical morbidities related to extensive surgery, several less aggressive treatment modalities have been proposed since the late 1970s. Photodynamic therapy is an effective and safe treatment for cutaneous non-melanoma skin cancer, with favorable cosmetic outcomes. Methods: in the present paper, the results of selected studies on photodynamic therapy in the treatment of vulvar intraepithelial neoplasia are reported and discussed. Results: Overall, complete histological response rates ranged between 20% and 67% and symptom response rates ranged between 52% and 89% according to different studies and case series. Conclusions: the real benefit of photodynamic therapy in the setting of vulvar intraepithelial neoplasia lies in its ability to treat multi-focal disease with minimal tissue destruction, preservation of vulvar anatomy and excellent cosmetic outcomes. These properties explain why photodynamic therapy is an attractive option for vulvar intraepithelial neoplasia treatment.

## 1. Introduction

Vulvar intraepithelial neoplasia (VIN) is a non-invasive precursor lesion usually found in 50–70% of patients affected by vulvar squamous cell carcinoma (VSCC) [1]. Squamous precursor lesions of the vulva were first identified about a century ago, and are characterized by cytological and architectural features of dysplasia, but without any sign of stromal invasion. Moreover, it is widely known that these intraepithelial lesions could precede the development of VSCC by a variable period [1].

There are two different pathogenetic pathways leading to VSCC, through the development of two distinct vulvar pre-cancerous lesions: (i) VIN of usual type (uVIN), also referred as classic or bowenoid VIN, that is associated to Human Papillomavirus (HPV) infection; and (ii) VIN of differentiated type (dVIN), also known as simplex VIN, that is HPV-independent [2,3].

Over time, various terms and many classification schemes have been proposed. The term “intraepithelial neoplasia” was first proposed by Richart in 1967 for lesions of the cervix, and only later for the vulva. In 1986, the International Society for the Study of Vulvo-Vaginal Disease (ISSVD) introduced the term VIN and identified three grades (VIN 1, 2, 3) according to the depth of dysplastic epithelial involvement [1,4]. An additional category, “VIN 3, differentiated type”, was defined [5]. Nevertheless, emerging evidence argued that there was not a biological continuum, as suggested by this classification. VIN 1 was usually represented by flat condylomatous lesions and associated with low-risk (LR) HPV genotypes, especially 6 and 11. On the contrary, VIN 2 and 3 were usually related to risk of progression to VSCC and associated with high-risk (HR) HPV genotypes [6]. Due to the negligible progression risk of VIN 1, in 2004, the ISSVD proposed a new classification, including (i) uVIN that comprehended lesions previously classified as VIN 2 and VIN 3; and (ii) dVIN [7].

More recently, a new consensus was reached by multiple committees, all supporting the terminology “squamous intraepithelial lesion”. In 2012, the Lower Anogenital Squamous Terminology (LAST) was proposed by the College of American Pathologists (CAP) and the American Society for Colposcopy and Cervical Pathology (ASCCP) in order to unify the terminology applied to HPV lesions affecting the cervix, vagina, vulva, perineum, anus and penis, under two categories: (i) low-grade squamous intraepithelial lesion (L-SIL); and (ii) high-grade squamous intraepithelial lesion (H-SIL) [8]. The 2014 World Health Organization (WHO) and the 2015 ISSVD classifications accepted the LAST, but included dVIN as an additional category [9].

The incidence of both uVIN, nowadays named vulvar H-SIL (vH-SIL), and dVIN has risen over the last three decades, even if the incidence of VSCC has remained relatively stable. Indeed, the incidence of vH-SIL almost doubled from 1.2/100,000 in 1992 to 2.1/100,000 women in 2005, whereas the incidence of dVIN even increased nine-fold from 0.013/100,000 to 0.121/100,000 women [10,11]. The rising number of VINs may reflect a real increased incidence related to the growing age of the population or a better recognition and more effective treatment of these precursor lesions before the development of VSCC [12].

Regarding aetiology, many agents, like herpes simplex virus (HSV), arsenic and granulomas, have been suggested as being responsible, before HPVs role was showed for the majority of anogenital squamous carcinomas, including VSCC [13]. HPV infection is strongly associated with vH-SIL, accounting for more than 80% of vH-SIL. HPV 16 is the most common genotype identified in vH-SIL and HPV-dependent VSCC, followed by other genotypes, such as 18, 31, 33 and 45 [14].

Lichen sclerosus (LS) has been suggested as a precursor of dVIN and HPV-independent VSCC, but the carcinogenic mechanism has not been established yet. The association was probably supposed because LS is a frequent finding in the skin adjacent to dVIN and VSCC, and because women with LS have a significantly higher risk of developing VSCC. Nevertheless, most of the women with LS do not develop dVIN or VSCC [15].

vH-SIL usually occurs in young women, in the third to the fifth decades, with the same risk factors as cervical lesions, such as number of sexual partners, smoking and immunosuppression [16]. vH-SIL appears as white, erythematous or pigmented macules or papules, that can coalesce to create plaques. Most patients report pruritus or pain, but almost 20% of women are asymptomatic [17]. Over half of patients have multifocal lesions of the vulva and present with multicentric lesions of the ano-genital tract, therefore a thorough examination is strongly recommended [18].

dVIN typically affects post-menopausal women in the sixth to the eight decades, and is often associated with LS or other chronic inflammatory dermatoses, such as lichen simplex chronicus. As opposed to vH-SIL, dVIN tends to be a single lesion and may appear as a grey-white stain, a thick plaque or a nodule. A long history of itching, burning and vulvar pain is reported by 60% of patients. However, many dVINs are asymptomatic and represent a large clinical challenge [17].

The risk of progression of vH-SIL to VSCC is 9–16% in untreated patients and about 3% in treated patients, respectively. Advanced age, previous radiotherapy and immunosuppression have been recognized as potential risk factors for malignant progression. Recurrence occurs in approximately 30% of patients, but the role of margin status and of new HPV acquisition have not been well defined [19]. Spontaneous regression has been reported in less than 1.5% of women, especially in patients younger than 35 years, pregnant women and those with multifocal lesions [20] (Table 1).

dVIN has higher risk of progression (32.8%) over a shortened time-period (22.8 versus 41.4 months) than vH-SIL [12].

However, since dVIN is an uncommon lesion, it should be assumed that dVIN is often underdiagnosed and probably represents a transient lesion that rapidly progresses to VSCC [19]. Furthermore, the diagnosis of isolated dVIN is rather rare due to an application problem of the diagnostic criteria by many pathologists 

In the past, extended vulvectomy was usually considered as the standard treatment for VIN. Considering the risk of the malignant progression of VIN and the important psychological and physical morbidities related to radical surgery, several less aggressive therapies have arisen since the late 1970s. Nowadays, therapeutic options for vH-SIL include local excision, laser ablation, topical imiquimod, cidofovir or 5-fluorouracil and photodynamic therapy [21].

Current prophylactic HPV vaccines represent a promising tool for the prevention of HPV pre-neoplastic and neoplastic ano-genital lesions. The broad diffusion of HPV vaccination is widely expected to result in a significant reduction of incidence of vH-SIL, especially in young women [22,23]. Furthermore, there are great expectations for the clinical response of patients affected by vH-SIL through therapeutic synthetic long-peptide vaccines against the HPV-16 oncoproteins E6 and E7 [24].

HPV-related malignancies are associated with a persistent HPV infection. The host immune response plays a crucial role in determining the clearance or persistence of both HPV infections and HPV-related VIN. In immune-compromised patients, i.e., renal transplant recipients, the malignant potential of vH-SIL is 50 times higher compared to the general population [25]. HPV produces several proteins among which the oncoproteins E6 and E7. E6 and E7 oncoproteins can interfere with crucial cell-cycle checkpoints. For instance, HPV E6 can lead to the dysfunction of the tumour suppressor gene p53 [26], while E7 can inactivate the retinoblastoma tumour suppressor gene (pRb), leading to the overexpression of p16^ink4a^ and p14^arf^ and proliferation of infected cells [27]. In most vH-SIL lesions immunohistochemistry is positive to p16^ink4a^ and p14^arf^, but negative to p53 [28]. The knowledge of the role of the oncoproteins E6 and E7 may lead to the development of new strategies to increase the immune response to prevent HPV-related malignancies.

**Figure 1:**
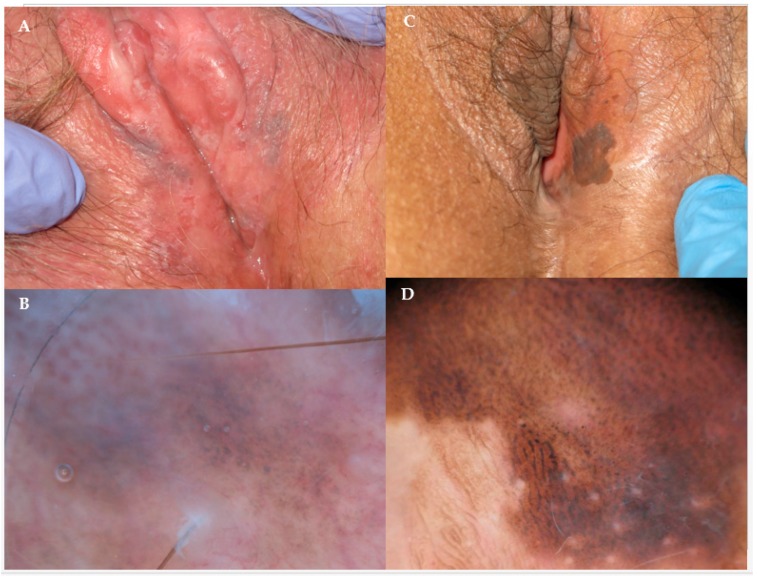
(**A**) Partially pigmented vulvar intraepithelial neoplasia (VIN) appearing as multiple slightly hyperkeratotic plaques, with erosions. (**B**) Digital dermoscopy revealed a brownish and pinkish background with some brown dots and irregularly distributed pigmented areas, a whitish veil and atypical vessels (original magnification ×40). (**C**) Pigmented, asymptomatic vulvar lesion presenting a rough surface and well demarcated borders. (**D**) Digital dermoscopy showed a brown background and hyperpigmented, cerebriform and fingerprint structures (original magnification ×40). All subjects gave their informed consent for inclusion before they participated in the study.

Screening tests for the diagnosis of VIN are not available. VIN is diagnosed during the visual assessment of the vulva. A tissue specimen can be obtained in order to have a pathologic confirmation. Recently, digital dermoscopy features of small case series of VIN lesions have been reported, suggesting that digital dermoscopy may become a useful diagnostic tool in the near future [29,30] (Figure 1).

## 2. Treatment of Vulvar Intraepithelial Neoplasia

VIN treatment is usually dictated by both the characteristics of the lesions and of patients. Lesion size, location, and the presence of a multifocal disease should be carefully considered together with patient’ age, comorbidities, psychological distress, symptoms and reliability for post-treatment follow-up. The availability of adequate medical resources and equipment is another key point which should be considered when planning VIN treatment [31]. The ideal treatment should completely ensure the absence of stromal invasion, improve subjective symptoms, reduce the risk of recurrence, and preserve vulvar function and morphology. To date, available treatments are not able to ensure these ideal results and the high morbidity associated with surgery makes it necessary to explore less harmful but effective approaches for VIN treatment [32].

dVIN is strongly associated with invasive squamous cell carcinoma (SCC) and surgical excision represents the treatment of choice for this condition. Full thickness tissue specimens obtained by surgery are appropriate for a complete and careful histological evaluation to evaluate the degree of stromal invasion. Medical therapies must be avoided in dVIN treatment [33].

Due to the increasing incidence of vH-SIL in younger women and the lower progression rate in this population a conservative and individualized management is indicated for vH-SIL, since extensive surgery may affect the body image and produce psychosexual distress.

Local excision, consisting of the removal of all visible lesions, can be performed with different techniques—scalpel, electrosurgery, or CO_2_ laser excision—with all techniques showing a similar efficacy. Several medical treatments have been used to avoid or limit surgery in vH-SIL patients. However, most of the studies lack of enough evidence because of the low number of subjects accrued, different inclusion criteria, comparison groups or limited follow-up [33]. Therefore, no medications are approved by the FDA for vH-SIL.

Two compounds with antiviral activity, the nucleoside analogue cidofovir, and the TLR7 agonist imiquimod, have been investigated as topical therapies in VIN patients. 

Cidofovir may induce the apoptosis of HPV-infected cells. In a pilot study of Tristram, 4 of the 10 patients treated with cidofovir 1% showed a complete response (CR). The side effects included ulceration at the site of application [34]. Cidofovir demonstrated response rates ranging from 40% to 79% in other small studies [35]. 

Imiquimod is a non-nucleoside heterocyclic amine, which acts as an immune-response modifier. Imiquimod induces the activity of interferon α (IFNα), tumor necrosis factor α (TNFα), and interleukin-6 via the stimulation of TLR7. Imiquimod demonstrated response rates ranging from 26% to 100% [36]. In the largest prospective, randomized, double-blinded, and placebo-controlled study with 52 patients, a 35% CR and 46% partial response (PR) were observed. After a median follow-up of 7.2 years, VIN recurred in only one of the patients who had experienced a CR, suggesting that imiquimod is effective in the long term [37]. 

The CRUK-funded RT3 VIN clinical trial randomized VIN 3 patients to therapy with either cidofovir or imiquimod. Histologically-proven CRs were observed in 41 of 72 (57%) cidofovir patients and 42 of 69 (61%) imiquimod patients [35]. 

A predictive biomarker that could identify patients likely to respond to specific treatments would facilitate the optimal management of these patients.

The strategy of vaccination using E6 and E7 HPV oncoproteins to treat VIN is attractive. However, studies for the development and research of vaccines are very expensive and the focus is placed on the study of vaccines to prevent lower genital tract neoplasia, rather than to treat it.

## 3. Photodynamic Therapy in the Treatment of Vulvar Intraepithelial Neoplasia

Topical photodynamic therapy (PDT) is an effective and safe treatment for cutaneous non-melanoma skin cancer (NMSC) with favorable cosmetic outcomes. The PDT technique involves the topical application of a photosensitizer, 5-aminolevulinic acid (ALA) or its methyl ester-methyl aminolevulinate (MAL), and subsequent illumination of the skin area with light of the appropriate wavelength. PDT is a useful nonsurgical treatment option for actinic keratosis (AK), superficial basal cell carcinoma (BCC) and in situ squamous cell carcinoma (SCC), especially at sites that are cosmetically sensitive or prone to impaired wound healing [38]. Besides NMSC, PDT is used to treat a wide range of medical conditions, including acne, skin aging, psoriasis, granuloma annulare, age-related macular degeneration and malignant neoplasms. The combination of photosensitizer, a light source and tissue oxygen leads to the chemical destruction of any tissues which have either selectively taken up the photosensitizer or have been locally exposed to light, with recruitment of inflammatory cells, increased immune response and vascular compromise. 

Similarly to the classical PDT used for the treatment of skin cancer, in order to achieve a selective destruction of the target vulvar areas, the photosensitizer agent can be applied topically onto the affected areas and subsequently locally excited with the proper light source. In some studies and case series aimed at the evaluation of PDT treatment for VIN, photosensitizers have been administered intravenously before delivering the light to the selected vulvar areas using an appropriate light source.

The different studies of PDT for VIN treatment are characterized by a non-standardized methodology regarding the type of photosensitiser, route of administration of the photosensitiser, duration of photosensitiser application, type and wavelength of the light source and number of treatment cycles per patient. Furthermore, and most importantly, the definition of treatment response was different.

The first study on the PDT treatment of VIN was published by Martin–Hirsch and colleagues in 1998. The photosensitiser used was 5-aminolaevulinic acid (ALA) and the light source was a non-laser light at a wavelength of 630 nm. The first 10 VIN patients received a single dose of 50 J cm^−2^, and two patients showed a histological response. After this first phase, eight women received a single treatment dose of 100 J cm^−2^, following which three out of eight women demonstrated a complete histological response. Pre-treatment analgesia was administered due to poor tolerability of the treatment. Sixteen out of the 18 women who received PDT treatment reported symptom relief (89%). Ten women were followed up for one to two years and nine of them developed local recurrence at the treatment site [39].

In a subsequent study, six patients received PDT treatment with 20% 5-ALA cream and a single dose of broad band light source 580–740 nm (single dose, 150 J cm^−2^). Five out of six women showed persistent disease at a six-month follow-up evaluation. Treatment was associated with erythema and erosions in the majority of cases [40].

In another study, 25 women were treated with topical 20% 5-ALA and 57 cycles of laser light at 635 nm wavelength (100 J cm^−2^). Thirteen (52%) patients with 27 VIN lesions achieved a complete histological response. Overall, 64% of the total 111 lesions treated regressed after one to three PDT courses. Hyperpigmented, hyperkeratotic and multifocal VIN lesions were negative predictive factors of response [41].

Fifteen women with high grade VIN received 10 g of 10% ALA gel followed with 120 J cm^−2^ laser light at a wavelength of 635 nm. The procedure was performed without anesthesia in most patients. Thirty patients with high-grade VIN treated by laser evaporation and 27 patients treated by surgical excision served as controls. Eight weeks following PDT, 11 of 15 patients obtained a histologically proven CR, with excellent tissue preservation. Three recurrences were recorded during follow-up, at five, six, and seven months after PDT. Twelve months after treatment, analysis of disease-free survival (DFS) revealed no statistically significant difference between patients treated with PDT and those treated with conventional modalities. In the multivariate analysis, multifocal disease was associated with a reduced DFS. The authors of this study concluded that PDT of high grade VIN showed the same efficacy as conventional treatment modalities, with excellent tissue healing and preservation [42].

Cases of high-grade VIN were enrolled in another study carried out at St. Mary’s Hospital in Manchester. Red light at 630 nm was used and 20% ALA cream was applied topically. At 12 weeks post-treatment, all patients were evaluated with a clinical inspection and histological confirmation. The first 10 VIN patients received a light dose of 50 J cm^−2^, and only two cases showed a short-term response. When the light dose was increased to 100 J cm^−2^, 8 of 22 (36.36%) patients showed normal histology at 12-week evaluation. The authors reported that small and unifocal lesions were most likely to respond, whereas hyperkeratinized and pigmented lesions were less responsive [43].

Another study evaluated the effect of the systemic photosensitizing agent meta-tetrahydroxyphenylchlorin (mTHPC) in high-grade VIN. In six patients a dose of 0.1 mg kg^−1^ body weight mTHPC was given intravenously and the area of VIN irradiated 96 h later with 652-nm light from a diode laser. Patients reported minimal pain from the initial treatment but two patients developed severe pain at the treated site for up to two weeks following PDT. All patients developed local oedema and one patient developed cellulitis. At six months two patients had developed VIN recurrence at the original site and one patient had a new VIN lesion. These were treated either with further PDT or with a small excision. At two years no VIN recurrence was recorded at the original site in all patients reviewed. In this small case series mTHPC-PDT was a useful treatment for high grade VIN, showing good cosmetic and functional outcomes, thus showing a major advantage over surgery [44].

In 2008, the results of a study on 20 women with high-grade VIN treated with topical imiquimod and PDT sequentially were published. The rationale for such a combination treatment was that topical imiquimod might elicit a local immune response and provide an enhanced immunological microenvironment for PDT to achieve a better clinical response than that previously documented from single agent regimens, with either imiquimod or PDT. Vulval biopsy and blood samples were taken pretreatment and after imiquimod and PDT, with follow up for one year. Biopsies were analyzed for HPV DNA and tumor-infiltrating lymphocytes including regulatory-T cells. The treatment was feasible for the majority of patients. There was an overall response rate of 55% by intention to treat and 64% per protocol. The 52-week symptom response was 65% asymptomatic, compared with 5% at baseline. Non-responder patients showed a significantly higher level of regulatory-T cells in the lesions after imiquimod treatment. Initial non-responders to imiquimod seem to be relatively refractory, and this may derive from their unfavorable local immune environment, in particular from the increased proportions of regulatory-T cells and possibly from the limiting action and/or development of any HPV T-cell immunity. The potential benefit of this treatment is its ability to treat multifocal disease [45].

In another study PDT was used for VIN patients using a novel bio adhesive patch to deliver aminolevulinic acid. Twenty-three patients underwent 49 cycles of PDT. Patches were designed to conform to uneven vulvar skin and contained 38 mg cm^−2^ aminolevulinic acid. Assessment was carried out at six weeks post-treatment. Fifty-two percent of patients reported a symptomatic response, with normal pathology restored in 38% of lesions. The patch was easy to apply and remove, causing minimal discomfort. Fluorescence inspection confirmed protoporphyrin accumulation. Pain during the procedure was problematic, necessitating some form of local analgesia. An analysis of changes in expression of apoptotic and cell cycle proteins (p53, p21, Mdm2, Blc-2, Bax, Ki-67) in response to PDT was evaluated. Treatment of VIN lesions using a bio adhesive patch induced changes in cell cycle and apoptotic proteins in response to PDT with the possible utilization of apoptotic pathways [46].

In a study published in 2015, fifteen patients received PDT for high-grade VIN (seven patients), vaginal intraepithelial neoplasia (VAIN, five patients), or vulvar Paget’s disease (three patients) between January 2003 and December 2013. Patients underwent colposcopy and/or vulvoscopy for assessment of lesions. Photoillumination with a 630-nm red laser light was applied to the lesions 48 h after intravenous injection of 2 mg kg^−1^ photosensitizer (PSZ; Photogem^®^). The light dose to the lesions was 150 J cm^−2^. Six out seven VIN patients experienced a CR which was maintained for at least 12 months, while in one case a persistent disease was recorded three months post treatment and the patients underwent partial vulvectomy. Regarding adverse events, photosensitivity reactions such as facial edema and urticaria occurred in 13.3% (2/15) and perineal pain occurred in one patient [47].

## 4. Photodynamic Therapy and Immune Response in Vulvar Intraepithelial Neoplasia

In the study by Abdel-Hady ES et al. [43], pre-treatment high-grade VIN biopsies were obtained from 19 of 32 patients. All of the 32 patients were biopsied at three months after PDT, and 11 cases had a second PDT after failure of the first treatment. HPV DNA was detected in 23 of 32 (72%) of the individual VIN specimens. Of the 10 women who were responders at the treatment site after PDT, four were HPV-negative before treatment, five became HPV-negative after treatment, and one remained HPV-positive. By contrast, of 22 non-responders, 17 had a persistent HR HPV infection. There was a greater likelihood of HPV positivity associated with a lack of response of VIN to PDT (*p* = 0.002). HLA expression was stable with no variation between pre- and post-treatment biopsies within the 19 cases that were analyzed. Interestingly, the 10 responding VINs did not show any HLA class I downregulation, whereas all VIN cases with total or allele loss did not respond and all but one had a HR HPV. Three out of six patients with total HLA class I loss developed microinvasive VSCC within one year. 

VIN lesions showed an immune infiltrate rich in in CD4 T cells and CD68+ cells but not CD1a Langerhans cells or CD8 T cells (CTLs) when compared with normal vulvar skin. However, post treatment, a significant increase of CD8 cells was found in VIN responders compared with non- responders (*p* = 0.0001). This study demonstrated that PDT was able to produce an immune response in which CD8 T cells were associated with a clinical and histological response, in complete accordance with previous animal-based models. Overall, these data supported the evidence that HR HPV infection and lack of cell-mediated immunity may be responsible for the observed poor response of VIN lesions to topical PDT [43].

Further investigation focused on the characterization of the T cell response in VIN showed a significantly higher number of total mean T lymphocytes in VIN specimens compared to normal vulval tissue (*p* = 0.002). In addition, in low-grade VIN there were significantly more CD8 cells than CD4 cells when compared to high-grade VIN. This difference in CD4/CD8 ratio was significant (*p* = 0.001) [48].

Combining microarray analysis with quantitative real-time polymerase chain reaction, it was shown that several chemokines were differentially expressed between VIN and control samples, with upregulation of IL8, CXCL10, CCL20 and CCL22 and down-regulation of CXCL12, CCL21 and CCL14). Furthermore, an increased number of mature dendritic cells (CD208+) seemed to be confined in the dermis, suggesting that these immune cells had not received the proper signal for migration to the lymph node, thus preventing them from producing an effective adaptive immune response and resulting in persistent HPV infection and an increased risk of developing cancer [49].

Taken together, these data clearly support the suggestion that the presence of a HR HPV infection and a diminished adaptive immune response play a key role in the outcome of PDT in VIN treatment [50].

## 5. Limits of Photodynamic Therapy in the Treatment of Vulvar Intraepithelial Neoplasia

Treatment failure in PDT is thought to be multifactorial, with insufficient tumour uptake of photosensitizer and PDT-induced suppression of antitumour responses potentially playing a key role in poor clinical outcomes [51,52]. Systemic and cutaneous antitumour immunity are crucial for skin cancer development and response to treatments. The immuno-suppressive effects of PDT have been reported [53]. 

The main disadvantage of PDT is that it can be painful and early termination of treatment due to poor tolerability may decrease the therapeutic efficacy of PDT [54]. Clinical experience showed that about 60% of patients could be treated without anesthesia, though temporary interruptions of the procedure were necessary in some cases. An effective analgesia using intravenous opioids or, sometimes, spinal or general anaesthesia can be considered and are advisable in some patients [55].

## 6. Conclusions

VIN is a non-invasive precursor lesion usually found in 50–70% of patients affected by vulvar squamous cell carcinoma (VSCC).

Two different pathogenetic pathways may lead to VSCC, through the development of two distinct vulvar pre-cancerous lesions: (i) VIN of usual type, also referred as classic or bowenoid VIN, now named vH-SIL, that is associated to HPV infection; and (ii) VIN of differentiated type (dVIN), also known as simplex VIN, that is HPV-independent. The incidence of both vH-SIL and dVIN has risen over the last three decades. In the past, extended vulvectomy was usually considered the standard treatment for VIN, however, considering the psychological and physical morbidities related to radical surgery, several less aggressive therapies have been proposed since the late 1970s. Among these approaches, PDT is an effective and safe treatment for cutaneous non-melanoma skin cancer, with favorable cosmetic outcomes. In the setting of VIN treatment, the different studies on PDT are characterized by a non-standardized methodology, regarding the type of photosensitiser, route of administration of the photosensitiser, duration of photosensitiser application, type and wavelength of the light source and number of treatment cycles per patient. Furthermore, and most importantly, the definition of treatment response was different.

In the present paper, the results of selected studies on PDT for VIN treatments have been reported. Overall, complete histological response rates ranged between 20% and 67% and symptom response rates ranged between 52% and 89% according to different studies and case series 

The real benefit of PDT in the treatment of VIN lies in its ability to treat multi-focal disease without tissue loss. Short healing time, minimal tissue destruction, preservation of vulval anatomy and excellent cosmetic outcomes are among the main advantages of PDT vs conventional surgical therapy [50]. These properties explain why PDT is an attractive option for VIN treatment.

## Figures and Tables

**Table 1:** Comparison of principal clinic, pathologic and prognostic characteristics between vulvar H-SIL (vH-SIL) and VIN of differentiated type (dVIN).

Characteristic	vH-SIL	dVIN
Age	Younger (third to fifth decades)	Older (sixth to eight decades)
Trigger	HPV infection	Lichen sclerosus and other chronic inflammatory dermatoses
Co-factors	Number of sexual partners, smoking, immunosuppression	Vulvar atypia, possibly mutated host genes
Multifocal lesions	Common	Rare
Symptoms	Pruritus or pain, asymptomatic in 20% of patients	Itching, burning and vulvar pain in 60% of patients, but often asymptomatic
Multicentric lesions of the ano-genital tract	Associated	Not associated
Risk of progression to VSCC	9–16%	32.8%
Median time of progression to VSCC (months)	41.4	22.8
